# *Adenomatous polyposis coli (APC)* gene promoter hypermethylation in primary breast cancers

**DOI:** 10.1054/bjoc.2001.1853

**Published:** 2001-07

**Authors:** Z Jin, G Tamura, T Tsuchiya, K Sakata, M Kashiwaba, M Osakabe, T Motoyama

**Affiliations:** Department of Pathology, Yamagata University School of Medicine, Yamagata 990-9585, Japan; Department of Surgery, Iwate Medical University of Medicine, Morioka 020-8505, Japan

**Keywords:** hypermethylation, *APC*, breast cancer, methylation-specific PCR

## Abstract

Similar to findings in colorectal cancers, it has been suggested that disruption of the adenomatous polyposis coli (APC)/β-catenin pathway may be involved in breast carcinogenesis. However, somatic mutations of *APC* and β- *catenin* are infrequently reported in breast cancers, in contrast to findings in colorectal cancers. To further explore the role of the *APC/β-catenin* pathway in breast carcinogenesis, we investigated the status of *APC* gene promoter methylation in primary breast cancers and in their non-cancerous breast tissue counterparts, as well as mutations of the *APC* and β- *catenin* genes. Hypermethylation of the *APC* promoter CpG island was detected in 18 of 50 (36%) primary breast cancers and in none of 21 non-cancerous breast tissue samples, although no mutations of the *APC* and β- *catenin* were found. No significant associations between *APC* promoter hypermethylation and patient age, lymph node metastasis, oestrogen and progesterone receptor status, size, stage or histological type of tumour were observed. These results indicate that *APC* promoter CpG island hypermethylation is a cancer-specific change and may be a more common mechanism of inactivation of this tumour suppressor gene in primary breast cancers than previously suspected. © 2001 Cancer Research Campaign http://www.bjcancer.com


British Journal of Cancer (2001) **85**(1), 69–73
© 2001 Cancer Research Campaign
doi: 10.1054/ bjoc.2001.1853, available online at http://www.idealibrary.com on IDEAL®

http://www.bjcancer.com

### *Adenomatous polyposis coli (APC)* gene promoter hypermethylation in primary breast cancers

**Z Jin[1], G Tamura[1], T Tsuchiya[1], K Sakata[1], M Kashiwaba[2], M Osakabe[1] and T Motoyama[1]**

[1]Department of Pathology, Yamagata University School of Medicine, Yamagata 990-9585, Japan; [2]Department of Surgery, Iwate Medical University of Medicine, Morioka 020-8505, Japan

**Summary** Similar to findings in colorectal cancers, it has been suggested that disruption of the adenomatous polyposis coli (APC)/β-catenin pathway may be involved in breast carcinogenesis. However, somatic mutations of *APC* and β-*catenin* are infrequently reported in breast cancers, in contrast to findings in colorectal cancers. To further explore the role of the *APC/β-catenin* pathway in breast carcinogenesis, we investigated the status of *APC* gene promoter methylation in primary breast cancers and in their non-cancerous breast tissue counterparts, as well as mutations of the *APC* and β-*catenin* genes. Hypermethylation of the *APC* promoter CpG island was detected in 18 of 50 (36%) primary breast cancers and in none of 21 non-cancerous breast tissue samples, although no mutations of the *APC* and β-*catenin* were found. No significant associations between *APC* promoter hypermethylation and patient age, lymph node metastasis, oestrogen and progesterone receptor status, size, stage or histological type of tumour were observed. These results indicate that *APC* promoter CpG island hypermethylation is a cancer-specific change and may be a more common mechanism of inactivation of this tumour suppressor gene in primary breast cancers than previously suspected. © 2001 Cancer Research Campaign http://www.bjcancer.com

**Keywords**: hypermethylation; *APC*; breast cancer; methylation-specific PCR

Mutations in the *adenomatous polyposis coli (APC)* tumour suppressor gene give rise to familial adenomatous polyposis and initiate the many, perhaps even majority of sporadic colon cancers (reviewed by Kinzler and Vogelstein, 1996). The observation that several copies of a 20-aminoacid repeat sequence in the central portion of the APC protein specifically associate with β-catenin was the first important clue in understanding the tumour-suppressive function of APC (Rubinfeld et al, 1993; Su et al, 1993). A quaternary cytoplasmic complex comprising APC, β-catenin, glycogen sythase kinase 3 β (GSK3 β) and axin leads to β-catenin phosphorylation by GSK3 β and, as a consequence, β-catenin is targeted for proteasomal destruction by ubiquitination (Hart et al, 1998; Nakamura et al, 1998). In colon carcinoma (Korinek et al, 1997; Morin et al, 1997) and melanoma cell lines (Rubinfeld et al, 1997), the nuclear accumulation of β-catenin caused by mutations in *APC* or *β-catenin* activate the transcription factor, T cell factor-4/lymphoid enhancer factor-1 (Tcf-4/Lef-1), thereby stimulating cell proliferation or inhibiting apoptosis. Thus the APC/β-catenin signaling pathway has been described to play a critical role in colorectal tumorigenesis (Korinek et al, 1997; Morin et al, 1997; Sparks et al, 1998). In addition, some studies have suggested the potential involvement of the Wnt-1 or APC/ β-catenin pathway in human breast cancers (Dale et al, 1996; Bui et al, 1997; Jonsson et al, 2000; Lin et al, 2000; Schlosshauer et al, 2000).

Recently, it has become apparent that tumour suppressor genes may be inactivated by aberrant DNA methylation of CpG islands in their promoter regions, including the *p16* (Herman et al, 1995; Gonzalgo et al, 1997), *E-cadherin* (Graff et al, 1995; Tamura et al, 2000), *BRCAI* (Mancini et al, 1998; Rice et al, 1998; Esteller et al, 2000a) and *hMLH1* (Kuismanen et al, 2000) genes. Altered DNA methylation in the *p16* (Herman et al, 1995), *E-cadherin* (Graff et al, 1995) and *BRCAI* genes (Mancini et al, 1998; Rice et al, 1998; Esteller et al, 2000a) has also been reported in breast cancers. In addition, *APC* promoter CpG island hypermethylation has been reported in colorectal (Hiltunen et al, 1997; Esteller et al, 2000b) and gastric cancers (Tsuchiya et al, 2000). Somatic mutations in *APC* have been reported only in 6% of primary breast cancers (Kashiwaba et al, 1994), despite frequent loss of heterozygosity (LOH) at the *APC* locus (Thompson et al, 1993; Medeiros et al, 1994; Kashiwaba et al, 1994) as well as frequent loss of APC protein expression (Ho et al, 1999). Therefore, we hypothesized that APC might be inactivated through promoter hypermethylation in primary breast cancers.

In order to test this hypothesis, we investigated the methylation status of *APC* gene promoter 1A (Esteller et al, 2000b; Tsuchiya et al, 2000) in DNAs from 50 breast cancers, as well as from 21 non-tumorous breast tissues by using the methylation-specific PCR (MSP) method. We also studied mutations of *APC* and β-*catenin* genes using PCR-single-stranded conformational polymorphism (SSCP) technique.

### MATERIALS AND METHODS

### Samples

50 cases of primary breast cancer surgically treated at the Department of Surgery, Iwate Medical University School of Medicine were included in this study (fresh non-tumorous tissue was not available for 29 cases). These tumors had been previously characterized for their stage, ER (oestrogen receptor) and PGR (progesterone receptor) using the EIA kits from Abbott

*Received 20 November 2000*
*Revised 15 March 2001*
*Accepted 27 March 2001*

*Correspondence to:* G Tamura

Diagnostics according to the manufacturer's instructions, and histology according to the histological classification of breast tumours of the Japanese Breast Cancer Society (1998). The samples were immediately frozen in liquid nitrogen after resection and stored at –80˚C until processing. The tumour and normal tissue samples were histologically confirmed. Genomic DNA was extracted using standard procedures (Sambrook et al, 1989).

### Methylation analysis

Genomic DNA extracted from 50 tumours and 21 non-tumorous samples was treated with sodium bisulfite as described previously (Clark et al, 1994). Two published primer-sets specific to sequences that correspond to either methylated or unmethylated DNA sequences of *APC* gene promoter 1A were used (Esteller et al, 2000b): (a) Mf 5′-TATTGCGGAGTGCGGGTC-3′ and Mr 5′-TCGACGAACTCCCGACGA-3′ for methylated DNA sequences of APC promoter; (b) UMf 5′-GTGTTTTATTGTGGAGT-GTGGGTT-3′ and Umr 5′-CCAATCAACAAACTCCCAACAA-3′ for unmethylated DNA sequences of APC promoter. Briefly, modified DNAs were amplified in a total volume of 20 ul 1 × GeneAmp PCR Gold Buffer (PE Applied Biosystems, Foster City, CA) containing 1.0 mM $MgCl_2$, 1 μM each primer, 0.2 mMμ dNTPs, and 1 unit Taq polymerase (AmpliTaq Gold DNA Polymerase, PE Applied Biosystems). After initial denaturation at 95˚C for 10 min, PCR was performed with 35 cycles consisting of denaturation at 95˚C for 15 second, annealing 60˚C for 15 second, and extension at 72˚C for 30 second, followed by a final 7 min extension step at 72˚C for two primer-sets. PCR products were then loaded onto non-denaturating 6% polyacrylamide gel, stained with ethidium bromide, and visualized under UV illumination. In addition, 100 μg of peripheral blood DNA as a positive control was treated according to the manufacturer's protocol (New England BioLabs, Inc., Beverly, MA) by Sss I Methylase. Sss I-treated DNA was modified by sodium bisulfite, amplified and electrophoresed as described above.

### DNA-sequencing analysis

The methylated and/or unmethylated MSP bands from 5 different tumours and 3 normal tissues were excised from gels and subjected to a second round of PCR amplification with the use of the same primers as used in the primary PCR. These PCR products were purified and sequenced using the dRhodamine Terminator Cycle Sequencing FS Ready Reaction Kit (PE Applied Biosystems) and an automated DNA sequencer (ABI PRISM 310, PE Applied Biosystems).

### Mutations analysis

PCR-SSCP analyses for codons 998–1141 and 1260–1547 of the *APC* gene, and exon 3 of the β-*catenin* gene were performed using published primer sequences and conditions (Kashiwaba et al, 1994; Park et al, 1999).

### Statistical analysis

Student's *t*-test and Fisher's exact probability test were performed for statistical analysis. A *P* value of less than 0.05 was considered statistically significant.

### RESULTS

Hypermethylation of *APC* promoter CpG islands was detected in 18 of 50 (36%) breast cancers. Unmethylated APC alleles were also present in all the 50 carcinomas (Figure 1A for representative results). In contrast, only unmethylated *APC* alleles were detected in the 21 corresponding non-cancerous breast tissues despite the presence of methylated *APC* alleles in 4 (20%) of their cancerous lesions (Figure 1B). The methylated and/or unmethylated MSP bands from 5 different tumours and 3 normal tissues were directly sequenced; the CpG islands in methylated band were always methylated (Figure 2A for representative results), while the CpG islands in unmethylated band were always unmethylated (Figure 2B). Neither *APC* nor β-*catenin* mutations were identified in any of the tumours by PCR-SSCP. There were no significant associations between *APC* promoter hypermethylation and patient age, stage, size or histological type of the tumour, lymph node metastasis, oestrogen or progesterone receptor status (Table 1).

### DISCUSSION

Our results demonstrate that *APC* promoter CpG island hypermethylation occurs frequently in primary breast cancers. The rate of hypermethylation of the *APC* promoter in our study (36%) is

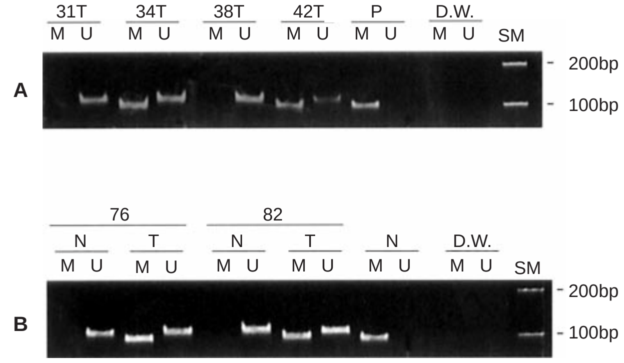

**Figure 1** Methylation analysis of *APC* promoter region in breast cancers. (**A**) methylated alleles are present in case 34 and 42, and only unmethylated alleles are seen in case 31 and 38 in their cancerous lesions; (**B**) methylated alleles are observed in the cancerous lesions but not in their corresponding non-cancerous tissues in case 76 and 82. T = cancerous lesion; N = non-cancerous tissue; M = methylated PCR products; U = unmethylated PCR products; P = positive control; D.W. = negative control; SM = size marker

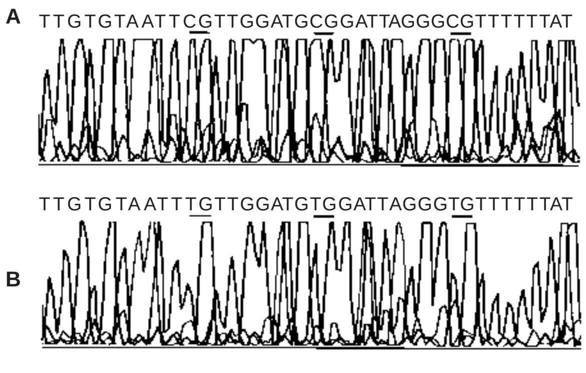

**Figure 2** Sequencing analysis of methylated and unmethylated *APC* alleles. (**A**) All CpG sites were methylated in the cancerous lesion of case 76; **B**, all CpG sites were unmethylated in the corresponding non-cancerous tissue of case 76, resulting in the sequence 'TpG' after bisulfite treatment

 *© 2001 Cancer Research Campaign*

**Table 1** Clinicopathological characteristics and methylation status of *APC* in breast cancers

| | **Number of tumours** | **Methylated status** | |
|---|---|---|---|
| | | **Methylated** | **Unmethylated** |
| Age (years) | | | |
| ≦40 | 6 | 2 | 4 |
| >40 | 40 | 15 | 25 |
| Unknown | 4 | 1 | 3 |
| Stage | | | |
| I | 10 | 6 | 4 |
| II | 26 | 7 | 19 |
| III | 9 | 4 | 5 |
| IV | 1 | 0 | 1 |
| Unknown | 4 | 1 | 3 |
| Histological type | | | |
| Invasive ductal carcinoma | 42 | 17 | 25 |
| Papillotubular carcinoma | 13 | 4 | 9 |
| Solid-tubular carcinoma | 5 | 2 | 3 |
| Scirrhous carcinoma | 24 | 11 | 13 |
| Noninvasive ductal carcinoma | 1 | 0 | 1 |
| Mucinous carcinoma | 2 | 0 | 2 |
| Invasive lobular carcinoma | 1 | 0 | 1 |
| Unknown | 4 | 1 | 3 |
| Tumour size | | | |
| ≦2.0 cm | 17 | 7 | 10 |
| >2.0 ≦5.0 cm | 20 | 7 | 13 |
| >5.0 cm | 8 | 2 | 6 |
| Unknown | 5 | 2 | 3 |
| Lymph node metastasis | | | |
| Positive | 26 | 9 | 17 |
| Negative | 20 | 8 | 12 |
| Unknown | 4 | 1 | 3 |
| Oestrogen receptor status | | | |
| Positive | 25 | 8 | 17 |
| Negative | 20 | 9 | 11 |
| Unknown | 5 | 1 | 4 |
| Progesterone receptor status | | | |
| Positive | 18 | 4 | 14 |
| Negative | 26 | 11 | 15 |
| Unknown | 6 | 3 | 3 |

similar to the rate of reduction of APC expression in a previous immunohistochemical study (40%) (Ho et al, 1999), which may explain the imbalance between LOH at the *APC* locus, APC protein expression and *APC* mutations (Thompson et al, 1993; Kashiwaba et al, 1994; Medeiros et al, 1994; Ho et al, 1999) in primary breast cancers. LOH studies have identified 5q, the region which includes the *APC* gene, as the site of loss in breast cancers, suggesting a possible role for *APC* in the progression of breast cancers (Thompson et al, 1993; Kashiwaba et al, 1994; Medeiros et al, 1994; Ho et al, 1999). It remains to be seen whether the hypermethylation we observed is associated with the silencing of gene expression; however, there are numerous examples in the literature of promoter hypermethylation strongly suppresses gene activity (Graff et al, 1995; Herman et al, 1995; Mancini et al, 1998; Rice et al, 1998; Esteller et al, 2000a, b; Kuismanen et al, 2000; Tamura et al, 2000; Tsuchiya et al, 2000). Among these reports, Esteller et al (Esteller et al, 2000b) demonstrated that *APC* promoter hypermethylation is biased toward tumours with genetically intact *APC* and associated with transcriptional silencing in colorectal cancers. Although *APC* promoter hypermethylation was previously detected in only one of 19 (5%) primary breast cancers (Esteller et al, 2000b) which was less frequent than our present data, this difference might be derived from difference of race or MSP sensitivity. It is also possible that it might simply reflect the difference of samples analysed. Our findings indicate that hypermethylation of *APC* promoter CpG islands may be a more common mechanism of inactivation of this tumour suppressor gene in primary breast cancers.

In breast cancers, the frequent LOH at the *APC* locus on chromosome 5q21 combined with the loss of APC protein expression (Thompson et al, 1993; Kashiwaba et al, 1994; Medeiros et al, 1994; Ho et al, 1999) and infrequent somatic mutations of the *APC* gene (Kashiwaba et al, 1994) are not suitable for the classic two-hit inactivation mechanism. In this context, Jones and Laird (Jones and Laird, 1999) set forth a new mechanism proposing that aberrant methylation of a promoter may be a ‘second hit’ accompanied with LOH or mutation for tumour suppressor gene inactivation. In recent studies in colorectal cancers, Esteller et al (Esteller et al, 2000b) demonstrated that both alleles of *APC* are functionally lost, one being deleted and the other methylated, as shown for the *VHL* gene in renal cell carcinoma (Herman et al, 1994), the *RBI* gene in retinoblastoma (reviewed by Jones and Laird, 1999), the *p16* gene in melanoma (Gonzalgo et al, 1997), and the *BRCA1* gene in breast cancer (Esteller et al, 2000a). In our study, hypermethylation of the *APC* promoter was detected in 18 of 50 (36%) of the breast cancers which was similar to the rate (38%) of the LOH study by Kashiwaba et al (Kashiwaba et al, 1994), and no mutations were found. We speculate that the rarity of observed

© 2001 Cancer Research Campaign 

somatic mutations of *APC* is due to the greater likelihood of APC inactivation by methylation in breast cancers.

In previous studies of colorectal and gastric cancers, methylation of the promoter CpG islands of *APC* was observed in cancerous as well as non-cancerous tissues (Hiltunen et al, 1997; Tsuchiya et al, 2000). Our study is the first to show that methylation of the promoter CpG island of *APC* occur specifically in breast cancers but not in their corresponding non-cancerous breast tissues. In addition, it has been shown that promoter CpG island methylation is related to aging in several genes, including the *ER* gene, the *insulin-like growth-factor-II* gene *(IGF2)* and others (reviewed by Issa, 2000). However, in our study, methylation of *APC* in breast cancers was not associated with the patient's age. Thus, hypermethylation of promoter CpG islands of *APC* is a cancer-specific change in the breast.

In the present study, we confirmed the findings of previous studies (Kashiwaba et al, 1994; Candidus et al, 1996; Jonsson et al, 2000; Schlosshauer et al, 2000) that the mutations of *APC* and/or β-*catenin* are infrequent in primary breast cancers. None of these studies analysed *APC* promoter CpG islands hypermethylation in the same samples, although some studies have indicated the potential involvement of the APC/β-catenin pathway in human breast cancers (Jonsson et al, 2000; Lin et al, 2000; Schlosshauer et al, 2000). In our series, no mutations in the *APC* and β-*catenin* genes were found, and *APC* promoter CpG islands hypermethylation was identified in 36% of breast cancers. Therefore, it is likely that methylation of *APC* gene may disrupt the regulation in the APC/β-catenin pathway in breast cancers.

In conclusion, hypermethylation of *APC* promoter CpG islands is a cancer-specific change, and may be a more common mechanism of inactivation of this tumour suppressor gene in primary breast cancers than previously thought.

### REFERENCES

Bui TD, Rankin J, Smith K, Huguet EL, Ruben S, Strachan T, Harris AL and Lindsay S (1997) A novel human Wnt gene, WNT10B, maps to 12q13 and is expressed in human breast carcinomas. *Oncogene* **14**: 1249–1253

Candidus S, Bischoff P, Becker KF and Hofler H (1996) No evidence for mutations in the α-and β-catenin genes in human gastric and breast carcinomas. *Cancer Res* **56**: 49–52

Clark SJ, Harrison J, Paul CL and Frommer M (1994) High sensitivity mapping of methylated cytosines. *Nucleic Acids Res* **22**: 2990–2997

Dale TC, Weber-Hall SJ, Smith K, Huguet EL, Jayatilake H, Gusterson BA, Shuttleworth G, O'Hare M and Harris AL (1996) Compartment switching of WNT-2 expression in human breast tumors. *Cancer Res* **56**: 4320–4323

Esteller M, Silva JM, Dominguez G, Bonilla F, Matias-Guiu X, Lerma E, Bussaglia E, Prat J, Harkes IC, Repasky EA, Gabrielson E, Schutte M, Baylin SB and Herman JG (2000a) Promoter hypermethylation and BRCA1 inactivation in sporadic breast and ovarian tumors. *J Natl Cancer Inst* **92**: 564–569

Esteller M, Sparks A, Toyota M, Sanchez-Cespedes M, Capella G, Peinado MA, Gonzalez S, Tarafa G, Sidransky D, Meltzer SJ, Baylin SB and Herman JG (2000b) Analysis of adenomatous polyposis coli promoter hypermethylation in human cancer. *Cancer Res* **60**: 4366–4371

Gonzalgo ML, Bender CM, You EH, Glendening JM, Flores JF, Walker GJ, Hayward NK, Jones PA and Fountain JW (1997) Low frequency of p16/CDKN2A methylation in sporadic melanoma: comparative approaches for methylation analysis of primary tumors. *Cancer Res* **57**: 5336–5347

Graff JR, Herman JG, Lapidus RG, Chopra H, Xu R, Jarrard DF, Isaacs WB, Pitha PM, Davidson NE and Baylin SB (1995) E-cadherin expression is silenced by DNA hypermethylation in human breast and prostate carcinomas. *Cancer Res* **55**: 5195–5199

Hart MJ, de los Santos R, Albert IN, Rubinfeld B and Polakis P (1998) Downregulation of β-catenin by human Axin and its association with the APC tumor suppressor, beta-catenin and GSK3 β. *Curr Biol* **8**: 573–581

Herman JG, Latif F, Weng Y, Lerman MI, Zbar B, Liu S, Samid D, Duan DS, Gnarra JR, Linehan WM and Baylin SB (1994) Silencing of the VHL tumor-suppressor gene by DNA methylation in renal carcinoma. *Proc Natl Acad Sci U S A* **91**: 9700–9704

Herman JG, Merlo A, Mao L, Lapidus RG, Issa JP, Davidson NE, Sidransky D and Baylin SB (1995) Inactivation of the CDKN2/p16/MTS1 gene is frequently associated with aberrant DNA methylation in all common human cancers. *Cancer Res* **55**: 4525–4530

Hiltunen MO, Alhonen L, Koistinaho J, Myohanen S, Paakkonen M, Marin S, Kosma VM and Janne J (1997) Hypermethylation of the APC (adenomatous polyposis coli) gene promoter region in human colorectal carcinoma. *Int J Cancer* **70**: 644–648

Ho KY, Kalle WH, Lo TH, Lam WY and Tang CM (1999) Reduced expression of APC and DCC gene protein in breast cancer. *Histopathology* **35**: 249–256

Issa JP (2000) CpG-island methylation in aging and cancer. *Curr Top Microbiol Immunol* **249**: 101–118

Jones PA and Laird PW (1999) Cancer epigenetics comes of age. *Nat Genet* **21**: 163–167

Jonsson M, Borg A, Nilbert M and Andersson T (2000) Involvement of adenomatous polyposis coli (APC) /β-catenin signalling in human breast cancer. *Eur J Cancer* **36**: 242–248

Kashiwaba M, Tamura G and Ishida M (1994) Aberrations of the APC gene in primary breast carcinoma. *J Cancer Res Clin Oncol* **120**: 727–731

Kinzler KW and Vogelstein B (1996) Lessons from hereditary colorectal cancer. *Cell* **87**: 159–170

Korinek V, Barker N, Morin PJ, van Wichen D, de Weger R, Kinzler KW, Vogelstein B and Clevers H (1997) Constitutive transcriptional activation by a β-catenin-Tcf complex in APC-/-colon carcinoma *Science* **275**: 1784–1787

Kuismanen SA, Holmberg MT, Salovaara R, de la Chapelle A and Peltomaki P (2000) Genetic and epigenetic modification of MLH1 accounts for a major share of microsatellite-unstable colorectal cancers. *Am J Pathol* **156**: 1773–1779

Lin SY, Xia W, Wang JC, Kwong KY, Spohn B, Wen Y, Pestell RG and Hung MC (2000) β-catenin, a novel prognostic marker for breast cancer: its roles in cyclin D1 expression and cancer progression. *Proc Natl Acad Sci U S A* **97**: 4262–4266

Mancini DN, Rodenhiser DI, Ainsworth PJ, O'Malley FP, Singh SM, Xing W and Archer TK (1998) CpG methylation within the 5′ regulatory region of the BRCA1 gene is tumor specific and includes a putative CREB binding site. *Oncogene* **16**: 1161–1169

Medeiros AC, Nagai MA, Neto MM and Brentani RR (1994) Loss of heterozygosity affecting the APC and MCC genetic loci in patients with primary breast carcinomas. *Cancer Epidemiol Biomarkers Prev* **3**: 331–333

Morin PJ, Sparks AB, Korinek V, Barker N, Clevers H, Vogelstein B and Kinzler KW (1997) Activation of β-catenin-Tcf signaling in colon cancer by mutations in β-catenin or APC *Science* **275**: 1787–1790

Nakamura T, Hamada F, Ishidate T, Anai K, Kawahara K, Toyoshima K and Akiyama T (1998) Axin, an inhibitor of the Wnt signalling pathway, interacts with β-catenin, GSK-3 β and APC and reduces the β-catenin level. *Genes Cells* **3**: 395–403

Park WS, Oh RR, Park JY, Lee SH, Shin MS, Kim YS, Kim SY, Lee HK, Kim PJ, Oh ST, Yoo NJ and Lee JY (1999) Frequent somatic mutations of the β-catenin gene in intestinal-type gastric cancer. *Cancer Res* **59**: 4257–4260

Rice JC, Massey-Brown KS and Futscher BW (1998) Aberrant methylation of the BRCA1 CpG island promoter is associated with decreased BRCA1 mRNA in sporadic breast cancer cells. *Oncogene* **17**: 1807–1812

Rubinfeld B, Souza B, Albert I, Muller O, Chamberlain SH, Masiarz FR, Munemitsu S and Polakis P (1993) Association of the APC gene product with β-catenin. *Science* **262**: 1731–1734

Rubinfeld B, Robbins P, El-Gamil M, Albert I, Porfiri E and Polakis P. (1997) Stabilization of β-catenin by genetic defects in melanoma cell lines *Science* **275**: 1790–1792

Sambrook J, Frisch EF and Maniatis T (1989) *Molecular cloning: a laboratory manual*, 2nd edn, Cold Spring Harbor Laboratory, New York

Schlosshauer PW, Brown SA, Eisinger K, Yan Q, Guglielminetti ER, Parsons R, Ellenson LH and Kitajewski J (2000) APC truncation and increased β-catenin levels in a human breast cancer cell line. *Carcinogenesis* **21**: 1453–1456

Sparks AB, Morin PJ, Vogelstein B and Kinzler KW (1998) Mutational analysis of the APC/β-catenin/Tcf pathway in colorectal cancer. *Cancer Res* **58**: 1130–1134

Su LK, Vogelstein B and Kinzler KW (1993) Association of the APC tumor suppressor protein with catenins. *Science* **262**: 1734–1737

Tamura G, Yin J, Wang S, Fleisher AS, Zou T, Abraham JM, Kong D, Smolinski KN, Wilson KT, James SP, Silverberg SG, Nishizuka S, Terashima M, Motoyama T

*© 2001 Cancer Research Campaign*

and Meltzer SJ (2000) E-Cadherin gene promoter hypermethylation in primary human gastric carcinomas. *J Natl Cancer Inst* **92**: 569–573

Thompson AM, Morris RG, Wallace M, Wyllie AH, Steel CM and Carter DC (1993) Allele loss from 5q21 (APC/MCC) and 18q21 (DCC) and DCC mRNA expression in breast cancer. *Br J Cancer* **68**: 64–68

Tsuchiya T, Tamura G, Sato K, Endoh Y, Sakata K, Jin Z, Motoyama T, Usuba O, Kimura W, Nishizuka S, Wilson KT, James SP, Yin J, Fleisher AS, Zou T, Silverberg SG, Kong D and Meltzer SJ (2000) Distinct methylation patterns of two APC gene promoters in normal and cancerous gastric epithelia. *Oncogene* **19**: 3642–3646

*© 2001 Cancer Research Campaign*

## References

[PDF_00318] Bui T. D., Rankin J., Smith K., Huguet E. L., Ruben S., Strachan T., Harris A. L., Lindsay S. (1997). A novel human Wnt gene, WNT10B, maps to 12q13 and is expressed in human breast carcinomas.. Oncogene.

[PDF_00321] Candidus S., Bischoff P., Becker K. F., Höfler H. (1996). No evidence for mutations in the alpha- and beta-catenin genes in human gastric and breast carcinomas.. Cancer Res.

[PDF_00324] Clark S. J., Harrison J., Paul C. L., Frommer M. (1994). High sensitivity mapping of methylated cytosines.. Nucleic Acids Res.

[PDF_00326] Dale T. C., Weber-Hall S. J., Smith K., Huguet E. L., Jayatilake H., Gusterson B. A., Shuttleworth G., O'Hare M., Harris A. L. (1996). Compartment switching of WNT-2 expression in human breast tumors.. Cancer Res.

[PDF_00329] Esteller M., Silva J. M., Dominguez G., Bonilla F., Matias-Guiu X., Lerma E., Bussaglia E., Prat J., Harkes I. C., Repasky E. A. (2000). Promoter hypermethylation and BRCA1 inactivation in sporadic breast and ovarian tumors.. J Natl Cancer Inst.

[PDF_00334] Esteller M., Sparks A., Toyota M., Sanchez-Cespedes M., Capella G., Peinado M. A., Gonzalez S., Tarafa G., Sidransky D., Meltzer S. J. (2000). Analysis of adenomatous polyposis coli promoter hypermethylation in human cancer.. Cancer Res.

[PDF_00338] Gonzalgo M. L., Bender C. M., You E. H., Glendening J. M., Flores J. F., Walker G. J., Hayward N. K., Jones P. A., Fountain J. W. (1997). Low frequency of p16/CDKN2A methylation in sporadic melanoma: comparative approaches for methylation analysis of primary tumors.. Cancer Res.

[PDF_00342] Graff J. R., Herman J. G., Lapidus R. G., Chopra H., Xu R., Jarrard D. F., Isaacs W. B., Pitha P. M., Davidson N. E., Baylin S. B. (1995). E-cadherin expression is silenced by DNA hypermethylation in human breast and prostate carcinomas.. Cancer Res.

[PDF_00346] Hart M. J., de los Santos R., Albert I. N., Rubinfeld B., Polakis P. (1998). Downregulation of beta-catenin by human Axin and its association with the APC tumor suppressor, beta-catenin and GSK3 beta.. Curr Biol.

[PDF_00349] Herman J. G., Latif F., Weng Y., Lerman M. I., Zbar B., Liu S., Samid D., Duan D. S., Gnarra J. R., Linehan W. M. (1994). Silencing of the VHL tumor-suppressor gene by DNA methylation in renal carcinoma.. Proc Natl Acad Sci U S A.

[PDF_00353] Herman J. G., Merlo A., Mao L., Lapidus R. G., Issa J. P., Davidson N. E., Sidransky D., Baylin S. B. (1995). Inactivation of the CDKN2/p16/MTS1 gene is frequently associated with aberrant DNA methylation in all common human cancers.. Cancer Res.

[PDF_00357] Hiltunen M. O., Alhonen L., Koistinaho J., Myöhänen S., Päkkönen M., Marin S., Kosma V. M., Jänne J. (1997). Hypermethylation of the APC (adenomatous polyposis coli) gene promoter region in human colorectal carcinoma.. Int J Cancer.

[PDF_00361] Ho K. Y., Kalle W. H., Lo T. H., Lam W. Y., Tang C. M. (1999). Reduced expression of APC and DCC gene protein in breast cancer.. Histopathology.

[PDF_00363] Issa J. P. (2000). CpG-island methylation in aging and cancer.. Curr Top Microbiol Immunol.

[PDF_00365] Jones P. A., Laird P. W. (1999). Cancer epigenetics comes of age.. Nat Genet.

[PDF_00367] Jönsson M., Borg A., Nilbert M., Andersson T. (2000). Involvement of adenomatous polyposis coli (APC)/beta-catenin signalling in human breast cancer.. Eur J Cancer.

[PDF_00370] Kashiwaba M., Tamura G., Ishida M. (1994). Aberrations of the APC gene in primary breast carcinoma.. J Cancer Res Clin Oncol.

[PDF_00372] Kinzler K. W., Vogelstein B. (1996). Lessons from hereditary colorectal cancer.. Cell.

[PDF_00374] Korinek V., Barker N., Morin P. J., van Wichen D., de Weger R., Kinzler K. W., Vogelstein B., Clevers H. (1997). Constitutive transcriptional activation by a beta-catenin-Tcf complex in APC-/- colon carcinoma.. Science.

[PDF_00377] Kuismanen S. A., Holmberg M. T., Salovaara R., de la Chapelle A., Peltomäki P. (2000). Genetic and epigenetic modification of MLH1 accounts for a major share of microsatellite-unstable colorectal cancers.. Am J Pathol.

[PDF_00380] Lin S. Y., Xia W., Wang J. C., Kwong K. Y., Spohn B., Wen Y., Pestell R. G., Hung M. C. (2000). Beta-catenin, a novel prognostic marker for breast cancer: its roles in cyclin D1 expression and cancer progression.. Proc Natl Acad Sci U S A.

[PDF_00384] Mancini D. N., Rodenhiser D. I., Ainsworth P. J., O'Malley F. P., Singh S. M., Xing W., Archer T. K. (1998). CpG methylation within the 5' regulatory region of the BRCA1 gene is tumor specific and includes a putative CREB binding site.. Oncogene.

[PDF_00388] Medeiros A. C., Nagai M. A., Neto M. M., Brentani R. R. (1994). Loss of heterozygosity affecting the APC and MCC genetic loci in patients with primary breast carcinomas.. Cancer Epidemiol Biomarkers Prev.

[PDF_00391] Morin P. J., Sparks A. B., Korinek V., Barker N., Clevers H., Vogelstein B., Kinzler K. W. (1997). Activation of beta-catenin-Tcf signaling in colon cancer by mutations in beta-catenin or APC.. Science.

[PDF_00394] Nakamura T., Hamada F., Ishidate T., Anai K., Kawahara K., Toyoshima K., Akiyama T. (1998). Axin, an inhibitor of the Wnt signalling pathway, interacts with beta-catenin, GSK-3beta and APC and reduces the beta-catenin level.. Genes Cells.

[PDF_00398] Park W. S., Oh R. R., Park J. Y., Lee S. H., Shin M. S., Kim Y. S., Kim S. Y., Lee H. K., Kim P. J., Oh S. T. (1999). Frequent somatic mutations of the beta-catenin gene in intestinal-type gastric cancer.. Cancer Res.

[PDF_00401] Rice J. C., Massey-Brown K. S., Futscher B. W. (1998). Aberrant methylation of the BRCA1 CpG island promoter is associated with decreased BRCA1 mRNA in sporadic breast cancer cells.. Oncogene.

[PDF_00407] Rubinfeld B., Robbins P., El-Gamil M., Albert I., Porfiri E., Polakis P. (1997). Stabilization of beta-catenin by genetic defects in melanoma cell lines.. Science.

[PDF_00404] Rubinfeld B., Souza B., Albert I., Müller O., Chamberlain S. H., Masiarz F. R., Munemitsu S., Polakis P. (1993). Association of the APC gene product with beta-catenin.. Science.

[PDF_00412] Schlosshauer P. W., Brown S. A., Eisinger K., Yan Q., Guglielminetti E. R., Parsons R., Ellenson L. H., Kitajewski J. (2000). APC truncation and increased beta-catenin levels in a human breast cancer cell line.. Carcinogenesis.

[PDF_00416] Sparks A. B., Morin P. J., Vogelstein B., Kinzler K. W. (1998). Mutational analysis of the APC/beta-catenin/Tcf pathway in colorectal cancer.. Cancer Res.

[PDF_00419] Su L. K., Vogelstein B., Kinzler K. W. (1993). Association of the APC tumor suppressor protein with catenins.. Science.

[PDF_00425] Tamura G., Yin J., Wang S., Fleisher A. S., Zou T., Abraham J. M., Kong D., Smolinski K. N., Wilson K. T., James S. P. (2000). E-Cadherin gene promoter hypermethylation in primary human gastric carcinomas.. J Natl Cancer Inst.

[PDF_00427] Thompson A. M., Morris R. G., Wallace M., Wyllie A. H., Steel C. M., Carter D. C. (1993). Allele loss from 5q21 (APC/MCC) and 18q21 (DCC) and DCC mRNA expression in breast cancer.. Br J Cancer.

[PDF_00430] Tsuchiya T., Tamura G., Sato K., Endoh Y., Sakata K., Jin Z., Motoyama T., Usuba O., Kimura W., Nishizuka S. (2000). Distinct methylation patterns of two APC gene promoters in normal and cancerous gastric epithelia.. Oncogene.

